# A dynamic relation between whole-brain white matter microstructural integrity and anxiety symptoms in preadolescent females with pathological anxiety

**DOI:** 10.1038/s41398-022-01827-y

**Published:** 2022-02-08

**Authors:** Nakul Aggarwal, Lisa E. Williams, Do P. M. Tromp, Daniel S. Pine, Ned H. Kalin

**Affiliations:** 1 Department of Psychiatry, University of Wisconsin-Madison, 6001 Research Park Boulevard, Madison, WI 53719 USA; 2 Section on Developmental and Affective Neuroscience, National Institute of Mental Health, Bethesda, MD 20814 USA

**Keywords:** Psychiatric disorders, Neuroscience

## Abstract

Pathological anxiety typically emerges during preadolescence and has been linked to alterations in white matter (WM) pathways. Because myelination is critical for efficient neuronal communication, characterizing associations between WM microstructure and symptoms may provide insights into pathophysiological mechanisms associated with childhood pathological anxiety. This longitudinal study examined 182 girls enrolled between the ages of 9–11 that were treatment-naïve at study entry: healthy controls (*n* = 49), subthreshold-anxiety disorders (AD) (*n* = 82), or meeting DSM-5 criteria for generalized, social, and/or separation ADs (*n* = 51), as determined through structured clinical interview. Anxiety severity was assessed with the Clinical Global Impression Scale and Screen for Child Anxiety and Related Emotional Disorders (SCARED). Participants (*n* = 182) underwent clinical, behavioral, and diffusion tensor imaging (DTI) assessments at study entry, and those with pathological anxiety (subthreshold-AD and AD, *n* = 133) were followed longitudinally for up to 3 additional years. Cross-sectional ANCOVAs (182 scans) examining control, subthreshold-AD, and AD participants found no significant relations between anxiety and DTI measurements. However, in longitudinal analyses of girls with pathological anxiety (343 scans), linear mixed-effects models demonstrated that increases in anxiety symptoms (SCARED scores) were associated with reductions in whole-brain fractional anisotropy, independent of age (Std. β (95% CI) = −0.06 (−0.09 to −0.03), *F*(1, 46.24) = 11.90, *P* = 0.001). Using a longitudinal approach, this study identified a dynamic, within-participant relation between whole-brain WM microstructural integrity and anxiety in girls with pathological anxiety. Given the importance of WM microstructure in modulating neural communication, this finding suggests the possibility that WM development could be a viable target in the treatment of anxiety-related psychopathology.

## Introduction

Anxiety is dimensional and, when extreme, becomes maladaptive and is pathological. Anxiety disorders (ADs) are among the most common childhood psychiatric illnesses, affecting up to 30% of youth [1]. In addition, numerous children have subclinical and persistent anxiety symptoms that do not meet DSM-5 criteria [2, 3]. Like children with ADs, these children also suffer considerably and are at increased risk to develop more significant stress-related psychopathology later in life [2, 4, 5]. Because anxiety is dimensional in nature, studying the full range of anxiety may provide insights into the factors that contribute to the varying degrees of distress and disability experienced by children with pathological anxiety. Understanding the factors underlying the development and expression of anxiety in young girls is of particular interest because after the transition to adolescence there is a two-fold increase in the prevalence of ADs in adolescent girls compared to boys that persists throughout the reproductive years [6–8].

White matter (WM) microstructure is highly relevant to adolescent development as adolescence is characterized by changes in WM pathways critical for effective neuronal communication [9–12]. While many studies have examined WM pathways in adults with high trait anxiety and ADs [13–23], considerably less work has examined WM in anxious youth [24–29]. Given evidence linking prefrontal-limbic pathways to anxiety [21, 25, 30–36], our prior work focused on the uncinate fasciculus (UF), the major WM pathway linking prefrontal regions to temporal lobe structures, including the amygdala and anterior hippocampus [37, 38]. These studies demonstrated anxiety-related reductions in UF fractional anisotropy (FA), a measure of WM microstructural integrity, in adults, preadolescent children, and preadolescent non-human primates (NHPs) [21, 25, 39]. Interestingly, our findings in preadolescent children and preadolescent NHPs suggest that the relation between UF FA and anxiety is present in males but not in females [25, 39].

To more comprehensively characterize WM in anxious girls, in the current study we used a longitudinal approach to assess within-participant relations between WM parameters and anxiety symptoms in preadolescent girls (enrolled ages 9–11). Additionally, because of the dimensional nature of anxiety, we included girls with a wide range of anxiety symptoms – controls (low anxiety), subthreshold-ADs (mild-moderate anxiety), and ADs (meeting DSM-5 AD criteria for generalized, social, and/or separation ADs). We first performed cross-sectional analyses comparing WM integrity between girls with ADs, subthreshold-ADs, and controls. Next, in girls with pathological anxiety (subthreshold-AD and AD), using repeated clinical and imaging assessments over a 3-year period, we assessed the longitudinal within-participant relation between anxiety symptoms and WM microstructure. We also assessed relations between WM microstructure with age and pubertal status, which were controlled for in the anxiety-focused analyses.

## Methods

### Participants

#### Recruitment and clinical assessment

A total of 182 preadolescent girls with varying levels of anxiety were enrolled between ages 9 and 11 and characterized using clinical, behavioral, developmental, and neuroimaging assessments. Children were recruited from the Madison metropolitan area via community and school advertisements and mass emails. Girls were interviewed with the Kiddie-Schedule for Affective Disorders and Schizophrenia Present and Lifetime version (K-SADS-PL) [40], administered either directly by or under the supervision of a trained PhD-level clinical psychologist or a psychiatrist. Review of audiotapes demonstrated acceptable reliability (Cohen κ > 0.80), and tape review (by DSP) continued throughout the study to maintain interviewer fidelity. Clinicians also rated overall anxiety severity using the Clinical Global Impression Scale-Severity (CGI-S) [41]. Diagnoses and CGI-S ratings were reviewed and confirmed in group discussions with all study clinicians. Included participants had never received treatment for anxiety or other psychiatric illness, were not treatment seeking, and were eligible for an MRI scan. Major exclusion criteria included psychotropic medication use, severe psychopathology in need of immediate treatment, and diagnoses of major depressive disorder, obsessive compulsive disorder, post-traumatic stress disorder, autism, bipolar disorder, or schizophrenia. Informed assent and consent were obtained from all participants and their parents, in accordance with the Institutional Review Board of the University of Wisconsin-Madison. Individuals were compensated for their time and effort.

**Fig. 1:**
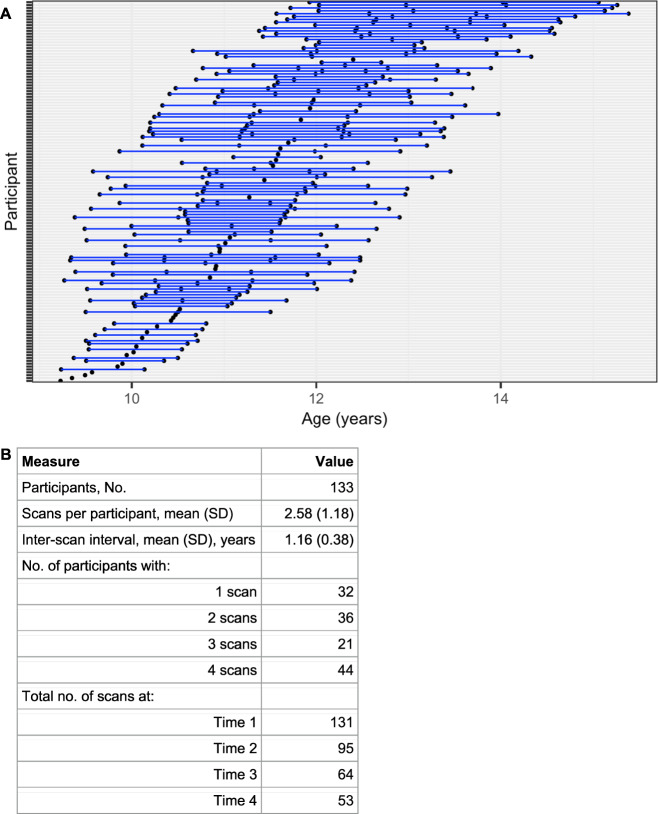
Depiction of the longitudinal study design. **A** Each blue line represents one participant; each point represents a scan for the respective participant and indicates age at scan. **B** Table with descriptive longitudinal scanning information.

Based on the K-SADS and CGI-S, participants were categorized into three groups: (1) healthy controls, (2) subthreshold-AD, and (3) AD. Healthy control participants exhibited very minimal, if any, symptoms of anxiety or any other psychiatric illness (CGI-S = 1, normal/not at all ill). Subthreshold-AD participants exhibited subsyndromal but persistent levels of symptoms associated with generalized, separation, and/or social anxiety but did not meet DSM-5 criteria for these disorders (CGI-S = 2, borderline mentally ill; or CGI-S = 3, mildly ill). A small number of girls in the subthreshold AD group (*n* = 5) met criteria for specific phobia. AD participants met full DSM-5 criteria for generalized anxiety disorder, separation anxiety disorder, and/or social anxiety disorder (CGI-S > 4, moderately ill or worse). A full description of the number of participants with each AD diagnosis or combination of AD diagnoses can be found in Table 1. Participants in the AD group could have comorbid diagnoses of attention deficit hyperactivity disorder and oppositional defiant disorder if symptoms were less severe than the AD. By design, girls with subthreshold-ADs did not meet full DSM-5 criteria for any disorders at study entry other than specific phobia. However, during longitudinal follow-up, 17 of the 82 girls (21%) in the subthreshold-AD group developed ADs or other psychiatric disorders. The final sample (*n* = 182) was comprised of 49 controls, 82 subthreshold-AD, and 51 AD girls who completed the initial year of the study. Girls with pathological anxiety (subthreshold-AD and AD, *n* = 133) were followed longitudinally for up to 3 years with annual clinical, neuroimaging, and behavioral assessments, including the K-SADS/CGI and multimodal imaging session. 131 participants were assessed in year 1, 95 in year 2, 64 in year 3, and 53 in year 4, for a total of 343 scans in the longitudinal sample (Fig. 1). There were no significant differences in anxiety severity at study entry (child SCARED) among children who completed the full study (all 4 scans) vs. those who completed either 1, 2, or 3 scans (Supplementary Fig. 1).

**Table 1:** Distribution of AD Cohort (*n* = 51) by Diagnoses.

Total GAD	18	Total SepAD	27	Total SocAD	24	Total Other-Specified AD	4
GAD only	5	SepAD only	14	SocAD only	11		
GAD + SepAD	4	SepAD + GAD	4	SocAD + GAD	4		
GAD + SocAD	4	SepAD + SocAD	4	SocAD + SepAD	4		
GAD + SepAD + SocAD	5	SepAD + GAD + SocAD	5	SocAD + GAD + SepAD	5		

*AD* anxiety disorder, *GAD* generalized anxiety disorder, *SepAD* separation anxiety disorder, *SocAD* social anxiety disorder.

#### Rating scales

Children’s anxiety symptoms were rated by both the child and a parent using the Screen for Child Anxiety and Related Emotional Disorders (SCARED) [42]. Depressive symptoms, environmental stressors, and externalizing behaviors were also assessed using the Child Depression Inventory (CDI) [43], Stressful Life Events Schedule (SLES) [44], and Conners’ Parent Rating Scale-Revised (CPRS-R) [45], respectively. The average interval between SCARED survey completion and MRI scan was 8.83 days (range: 0–178 days). Children’s pubertal status was measured with the Tanner Staging Scale and the Pubertal Development Scale (PDS), completed by child and parent together [46]. These data were collected and managed using REDCap (Research Electronic Data Capture) tools hosted at the University of Wisconsin-Madison, School of Medicine and Public Health, a secure, web-based application designed to support data capture for research studies [47, 48].

### MRI data acquisition and processing

#### DTI acquisition

All brain images were collected on a 3.0 Tesla GE MR750 scanner (GE Healthcare; Waukesha, WI) using a 32-channel head coil. Diffusion-weighted MRI scans were obtained using a two-dimensional echo planar imaging diffusion-weighted spin-echo sequence with 48 optimal non-collinear directions (see Supplementary Methods).

#### DTI processing and analysis

Image processing was completed using procedures described previously in Tromp et al., 2019 [25], with an additional step of within-participant co-registration of tensor images prior to co-registration across participants. Deterministic tractography was performed in TrackVis [49] to delineate whole-brain WM and seven bilateral tracts of interest across the brain. The 7 WM tracts were selected based on previous literature implicating alterations in these tracts in ADs and other internalizing disorders, including the UF [21, 25, 39], cingulum bundle (CING) [14, 26], superior longitudinal fasciculus (SLF) [13, 29], stria terminalis/fornix (STRIA/FX) [15], inferior fronto-occipital fasciculus (IFO) [29, 50], internal capsule (IC) [29, 51], and corpus callosum (CC) [17, 26]. For each image, a weighted mean was calculated per tract for each diffusion metric (fractional anisotropy [FA], mean diffusivity [MD], radial diffusivity [RD]) (see Supplementary Methods).

To identify potential anxiety-related WM alterations in regions outside the 7 pre-determined WM tracts, whole-brain voxel-wise FA analyses were performed both cross-sectionally (with all data collected at study entry) and longitudinally (with data from subthreshold-AD and AD participants), using the randomize program in FSL [52] and the 3dLMEr program in AFNI [53], respectively. Input data for both analyses consisted of normalized FA images in standard MNI space (i.e., 182 and 343 FA images included in the cross-sectional and longitudinal analyses, respectively).

### Statistical analysis

Cross-sectional analyses (*n* = 182 scans) assessed group differences in WM microstructure and behavioral metrics in the full initial sample of participants (controls vs. subthreshold-AD vs. AD). One-way ANCOVA models assessed between-group differences in: (1) demographic and clinical variables and (2) DTI metrics in the 7 WM tracts and whole-brain WM. Because child- and parent-rated SCARED scores were significantly correlated (see Supplementary Results), and because there is some evidence to suggest that child self-reports may be particularly relevant during this developmental period [54–56], child-rated SCARED scores were selected as the primary metric of anxiety severity. Analyses were also performed with parent-rated SCARED scores and are reported below. Linear regression models assessed the between-participant relationship between anxiety levels (child SCARED) and DTI metrics. Because age, PDS scores, and Tanner Staging scores were significantly inter-correlated (see Supplementary Results), age was used as the developmental covariate for all ANCOVA and regression models. Similar analyses substituting PDS or Tanner scores in place of age did not change the results (Supplementary Fig. 2). All models were evaluated using both frequentist and Bayesian statistics, the latter of which affords the ability to claim evidence of absence of an effect (see Supplementary Methods) [57]. Analyses were conducted using the stats and base packages in RStudio (ver. 1.4.1106) and JASP software (ver. 0.14.1). In the cross-sectional voxel-wise analysis, general linear models (using permutation methods [58]) were implemented with the FSL randomise tool [52] to estimate the relationship between child SCARED scores while controlling for age. Using threshold-free cluster enhancement (TFCE) [59], results were assessed at a family-wise error (FWE)-corrected threshold of *P* < 0.05.

Across the sample of girls with pathological anxiety (subthreshold-AD and AD participants; *n* = 343 scans), longitudinal within-participant relationships between age, child SCARED scores, and WM microstructure in the 7 WM tracts of interest and whole-brain were assessed using linear mixed-effects (LME) models, which allow for precise and unbiased effect estimates by accounting for repeated within-participant measures [60]. As in the cross-sectional analysis, within the longitudinal data age, PDS scores, and Tanner Staging scores were highly inter-correlated (see Supplementary Results), and age was used as the developmental covariate. Separate LME models quantified the within-participant relationship between: (1) age and child SCARED scores; and (2) child SCARED scores and WM microstructure in each tract, while controlling for age (see Supplementary Methods). LME modeling was performed using the lme4 and car packages in RStudio (ver. 1.4.1106) with frequentist statistics. Tractography-based DTI analyses used a Bonferroni-adjusted *P* value for multiple comparison correction (8 comparisons across 7 tracts and whole-brain WM; *P*_corrected_ < 0.05/8 = 0.00625). Within the longitudinal sample of girls with pathological anxiety, LME modeling was also performed at the whole-brain voxel-wise level with the 3dLMEr package in AFNI [53], estimating the within-participant relationship between child SCARED scores and FA in each voxel while controlling for age. Results were assessed at both the FDR-corrected level and at an uncorrected threshold of *P* < 0.005.

## Results

### Cross-sectional analysis (controls vs. subthreshold-AD vs. AD)

Groups did not differ in any demographic variable, except PDS scores (subthreshold-AD > control) (Table 2). As expected, groups differed on multiple clinical measures, in most cases in a stepwise manner (AD > sub-threshold-AD > control) (Table 2). For categorical (control vs. subthreshold-AD vs. AD) or dimensional (child SCARED) analyses, there were no statistically significant associations between anxiety and FA in the WM tracts of interest or in whole-brain WM after multiple comparison correction (Table 3). Bayesian analyses support the interpretation of these null effects as the absence of a relation between anxiety and FA in the CC, CING, IC, IFO, STRIA/FX, and UF (BFs_H1_ < 0.33), and a lack of evidence for anxiety-FA relations in the SLF and whole-brain WM (0.33 < BFs_H1_ < 3). As expected, age was associated with FA at the whole-brain level, as well as in the CING, IC, IFO, and UF, but not in the CC, SLF, or STRIA/FX (Supplementary Fig. 3 and Supplementary Table 1). Additionally, voxel-wise analyses did not show any significant FWE-corrected clusters in which child SCARED scores predicted FA.

**Table 2:** Sample characteristics by cohort at study entry.

Clinical Measure	Healthy Control (*n* = 49)	Subthreshold-AD (*n* = 82)	AD (*n* = 51)	One-Way ANOVA *P* value
Age, mean (SD), years	10.43 (0.82)	10.50 (0.85)	10.63 (0.78)	0.489
IQ (WASI), mean (SD)	113.92 (12.58)	116.77 (16.36)	113.94 (14.11)	0.449
PDS scores, mean (SD)	1.61 (0.50)	1.91 (0.55)	1.78 (0.58)	0.010^a^
Tanner Staging scores, mean (SD)	1.64 (0.76)	1.80 (0.74)	1.82 (0.93)	0.446
Parent SCARED (Anxiety), mean (SD)	3.04 (2.98)	18.93 (9.17)	31.02 (10.50)	<0.001^b^
Child SCARED (Anxiety), mean (SD)	6.92 (5.71)	23.65 (10.10)	33.40 (13.12)	<0.001^b^
CGI-S (Global Impression), mean (SD)	1.00 (0.00)	2.45 (0.50)	4.16 (0.46)	<0.001^b^
CDI (Depression), mean (SD)	40.40 (3.85)	44.74 (5.71)	50.02 (8.86)	<0.001^b^
ACE-Related SLES (Life Stressors) Count, mean (SD)	1.57 (1.89)	3.10 (2.85)	3.63 (3.57)	0.001^c^
CPRS (ADHD), mean (SD)	45.40 (3.75)	55.25 (9.98)	58.90 (11.98)	<0.001^d^

*WASI* Wechsler abbreviated scale of intelligence, *PDS* pubertal development scale, *SCARED* screen for child anxiety related emotional disorders, *CGI-S* clinical global impression scale-severity, *CDI* child depression inventory, *ACE* adverse childhood experiences, *SLES* stressful life events schedule, *CPRS-R* Conners’ parent rating scale-revised.

^a^Significant main effect of group (*P* < 0.05) in one-way ANOVA. Post-hoc Tukey indicates subthreshold-AD > controls (*P* = 0.007).

^b^Significant main effect of group (*P* < 0.001) in one-way ANOVA. Post-hoc Tukey indicates stepwise progression (controls < subthreshold-AD < AD; all *P* < 0.001).

^c^Significant main effect of group (*P* = 0.001) in one-way ANOVA. Post-hoc Tukey indicates subthreshold-AD and AD groups do not differ, but both are higher than controls (*P* < 0.05).

^d^Significant main effect of group (*P* < 0.001) in one-way ANOVA. Post-hoc Tukey indicates subthreshold-AD and AD groups do not differ, but both are higher than controls (*P* < 0.001).

**Table 3:** Group differences in tract FA and dimensional relations with SCARED scores at study entry^a^.

		FA, mean (SD)			One-Way ANCOVA		Linear Regression - Child SCARED	
Bilateral WM Tract		Control	Risk	AD	*P* value	BF (H_1_)	*P* value	BF (H_1_)
	WB	0.358 (0.009)	0.362 (0.010)	0.363 (0.008)	0.015	2.406	0.054	1.259
	CC	0.464 (0.013)	0.466 (0.015)	0.469 (0.012)	0.249	0.190^b^	0.329	0.370
	CING	0.315 (0.018)	0.319 (0.024)	0.320 (0.016)	0.523	0.101^b^	0.260	0.375
	IC	0.450 (0.012)	0.453 (0.013)	0.455 (0.012)	0.147	0.308^b^	0.054	0.829
	IFO	0.417 (0.013)	0.420 (0.015)	0.422 (0.012)	0.343	0.143^b^	0.773	0.232^b^
	SLF	0.395 (0.016)	0.400 (0.017)	0.404 (0.016)	0.030	1.214	0.048	0.694
	STRIA/FX	0.312 (0.015)	0.314 (0.016)	0.314 (0.015)	0.776	0.072^c^	0.865	0.249^b^
	UF	0.355 (0.013)	0.360 (0.013)	0.359 (0.014)	0.186	0.254^b^	0.930	0.230^b^

*CC* corpus callosum, *CING* cingulum, *IC* internal capsule, *IFO* inferior fronto-occipital fasciculus, *SLF* superior longitudinal fasciculus, *STRIA/FX* stria terminalis/fornix, *UF* uncinate fasciculus, *WB* whole-brain WM.

^a^All analyses control for age at scan. 3-D renderings (right sagittal views) generated from deterministic tractography are shown for each WM tract of interest and whole-brain WM. Green fibers extend along anterior-posterior axis; red fibers along the medial-lateral axis; and blue fibers along the superior-inferior axis.

^b^Moderate evidence of absence of an effect under the Bayesian framework (0.1 < BF_H1_ < 0.33).

^c^Strong evidence of absence of an effect under the Bayesian framework (BF_H1_ < 0.1).

### Longitudinal analysis in girls with pathological anxiety

**Fig. 2:**
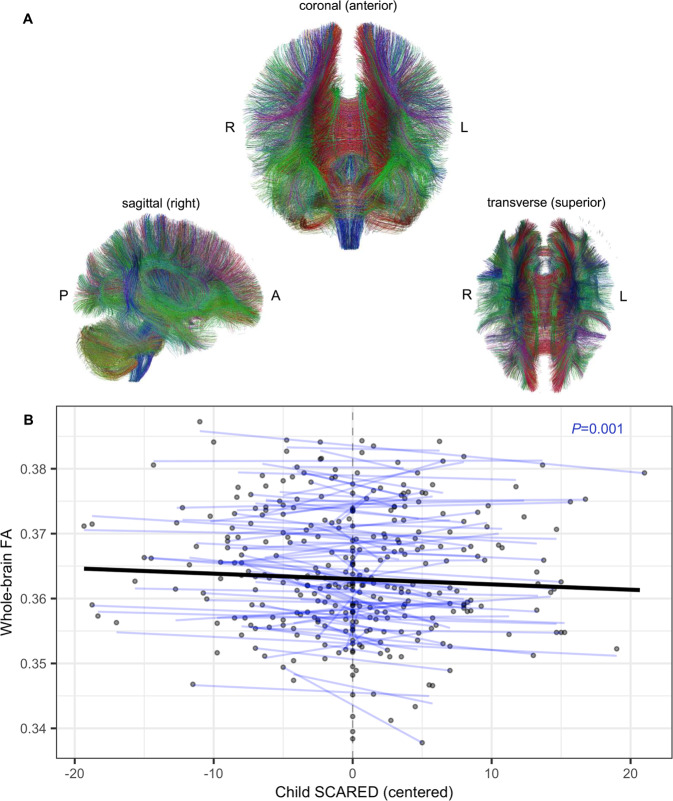
Longitudinal within-participant association of whole-brain FA with child SCARED scores. **A** Coronal, sagittal, and transverse views of a 3-D rendering of whole-brain WM tracts, as generated via deterministic tractography in TrackVis (A-P anterior-posterior; R-L right-left). Green fibers extend along anterior-posterior axis; red fibers along the medial-lateral axis; and blue fibers along the superior-inferior axis. **B** Within-participant relationship between whole-brain FA and child SCARED scores. Each blue line represents a participant-specific regression line predicting whole-brain FA from within-participant centered child SCARED scores, while controlling for age. Each point represents an individual scan. The bolded black line depicts the average within-participant association of whole-brain FA with child SCARED scores.

In our longitudinal sample of girls with subthreshold-ADs or ADs (*n* = 133), we examined the within-participant relations between changes in the severity of anxiety symptoms and associated WM microstructural changes throughout the brain while controlling for age. Child SCARED scores exhibited a significant negative correlation with whole-brain FA at the individual level (Std. β (95% CI) = −0.06 (−0.09 to −0.03), *F*(1, 46.24) = 11.90, *P* = 0.001), such that increases in a child’s anxiety level predicted decreases in her whole-brain FA (Table 4 and Fig. 2). While not statistically significant after multiple comparison correction, parallel analyses with MD and RD demonstrated consistent results (Supplementary Fig. 4 and Supplementary Table 2). Parallel analyses in each of the 7 WM tracts of interest did not reveal any statistically significant anxiety-FA associations after multiple comparison correction (Table 4). However, at the uncorrected level, multiple WM tracts – including the CC, CING, IFO, and SLF – showed reductions in FA in relation to anxiety severity (Table 4). Substituting parent SCARED scores for child SCARED scores in this analysis also revealed a negative within-participant association between anxiety and whole-brain FA but did not reach statistical significance (*P* = 0.18) (Supplementary Fig. 5). To complement these tract-based analyses, we also performed a voxel-wise analysis across the entire brain. Voxel-wise analyses did not reveal any significant FDR-corrected clusters in which child SCARED scores predicted FA. At an uncorrected threshold (*P* < 0.005), a number of small clusters (<120 voxels) distributed across the brain were negatively associated with child SCARED scores.

**Table 4:** Average within-participant associations between SCARED scores and tract FA^a^.

Bilateral WM Tract		β1	Std. β1 (95% CI)	*P* value
	WB	−8.438E-05	−0.06 (−0.09 to −0.03)	0.001^b^
	CC	−1.057E-04	−0.05 (−0.08 to −0.02)	0.007
	CING	−1.027E-04	−0.03 (−0.06 to −0.01)	0.034
	IC	−4.460E-05	−0.02 (−0.06 to 0.01)	0.220
	IFO	−1.072E-04	−0.05 (−0.08 to −0.02)	0.007
	SLF	−7.819E-05	−0.03 (−0.07 to 0.00)	0.044
	STRIA/FX	−8.780E-05	−0.04 (−0.08 to 0.00)	0.065
	UF	−4.753E-05	−0.02 (−0.06 to 0.02)	0.345

*CC* corpus callosum, *CING* cingulum, *IC* internal capsule, *IFO* inferior fronto-occipital fasciculus, *SLF* superior longitudinal fasciculus, *STRIA/FX* stria terminalis/fornix, *UF* uncinate fasciculus, *WB* whole-brain WM.

^a^All analyses control for age at scan. 3-D renderings (right sagittal views) generated from deterministic tractography are shown for each WM tract of interest and whole-brain WM. Green fibers extend along anterior-posterior axis; red fibers along the medial-lateral axis; and blue fibers along the superior-inferior axis.

^b^Statistically significant under the frequentist framework at a Bonferroni-corrected level (*P* < 0.00625).

While all participants were treatment-naïve at study entry, a subset (*n* = 27) began receiving behavioral and/or pharmacological therapy in follow-up years of the study. In a supplemental analysis, excluding scans collected after treatment initiation did not alter the findings (Supplementary Fig. 6). Supplementary analyses that included depression and stressful life events as within-participant covariates in separate models did not change the overall pattern of results (see Supplementary Results). While there was a positive within-participant relationship between age and whole-brain FA (Std. β (95% CI) = 0.08 (0.05 to 0.12), *F*(1, 64.90) = 24.75, *P* < 0.001) – in line with the cross-sectional analysis – there was no significant correlation between age and child SCARED scores (Supplementary Fig. 7 and Supplementary Table 3).

## Discussion

This study in young females is one of the largest longitudinal neuroimaging studies of pathological anxiety, focused on understanding alterations in neural pathways relevant to the development of anxiety during childhood. The major finding from this study was derived from the longitudinal data, revealing that within participants, more severe anxiety symptoms were associated with lower whole-brain FA (Table 4 and Fig. 2). This relation manifested in treatment-naïve girls, including girls with subthreshold AD symptoms as well as girls who met criteria for ADs, independent of age and pubertal status. We also performed cross-sectional analyses with data collected at study entry, comparing WM integrity among controls, subthreshold-AD participants, and AD participants. In contrast to the longitudinal finding, this cross-sectional analysis revealed no significant relations between FA and anxiety. Taken together, these findings highlight a dynamic relation between whole-brain WM integrity and anxiety, as well as the importance of a longitudinal within-participant approach for studying developmental psychopathology.

The presence of a significant longitudinal association between anxiety and WM in the context of no significant cross-sectional association is notable (Tables 1, 3). This suggests that within an individual, whole-brain FA and anxiety fluctuate together over time, regardless of individual differences in FA magnitude. WM microstructural integrity is influenced by genetic, experiential, and environmental factors. Given the sensitivity of WM to these variables, it is possible that between-subject variation in FA, which is typically greater than within-subject variation, could be due to differences in the extent to which children are impacted by factors that influence myelin development. This could account for our lack of a between-participant finding in the presence of a within-participant, longitudinal finding. We are unaware of other studies that have concomitantly examined the relation between anxiety symptoms and WM parameters longitudinally in children with pathological anxiety. One longitudinal DTI study in a normative sample of youth (ages 6–18) found that children with higher anxiety/depression symptoms at study intake had slower rates of WM development in multiple WM tracts [27]. Another study in a large sample of youth reported an association between initially assessed internalizing and externalizing symptoms with reduced growth-related increases in global WM [61]. Other work examining WM alterations in relation to pediatric anxiety has been cross-sectional. Four cross-sectional DTI studies have been performed in youth (ages 6–18), examining typically developing youth with trait anxiety as well as children with ADs [25, 26, 28, 29]. Consistent with DTI studies of anxiety in adults [13, 16, 17, 19], these studies have generally reported anxiety-related WM reductions in FA in various regions, including the UF, CING, CC, and IFO. We previously reported a reduction in UF FA in boys with ADs but not girls, also using a cross-sectional approach [25]. The lack of a relation between anxiety and UF FA in girls in our previous study is consistent with the cross-sectional results reported here in preadolescent girls with pathological anxiety. These null effects should be interpreted cautiously. We note that a Bayesian analysis performed on the data from the girls in the previous study did not support evidence for the absence of an effect (see Supplementary Results), whereas a Bayesian analysis performed on the current dataset was supportive of evidence of absence for the lack of an association between UF FA and anxiety (Table 3).

The correlation between global WM microstructure and anxiety symptoms suggests the presence of a diffuse whole-brain WM effect. Such an effect could have consequences for the inter- and intra-connectivity among brain networks relevant to emotional information processing and integration, aversive stimulus detection, and the interpretation of social behavior. Indeed, similar whole-brain WM microstructural alterations have been reported in relation to general psychopathology factors and cognitive abilities in youth [24, 62]. The tractography-based findings and whole-brain voxel-wise analysis are consistent with a distributed pattern of alterations in structural connectivity related to changes in anxiety on an individual level. This finding in girls with pathological anxiety contrasts with our previous report of UF-specific reductions in WM integrity in anxious boys, which implicates PFC-limbic alterations in pathological anxiety. The absence of this structural finding in girls does not preclude the possibility that functional studies might reveal other PFC-limbic alterations.

While our results suggest a dynamic relationship between WM microstructure and childhood anxiety, the mechanism underlying this association is unclear. Furthermore, it is possible that the association between WM integrity and anxiety symptoms is not causally linked, as other factors could concomitantly impact both of these measures. However, studies in both NHPs and humans suggest that stress can affect WM microstructure [63, 64]. As such, it is plausible that in our sample of girls, the experience of chronically heightened anxiety could result in altered WM microstructure. Specific mechanisms that have been implicated from preclinical studies link adversity to WM microstructure via effects on oligodendrogenesis and myelination [65, 66]. It is also conceivable that WM microstructure plays a role in directly mediating levels of anxiety. Studies manipulating oligodendrocyte function in preclinical models of anxiety could be informative in this regard. Our results provide an impetus to examine the potential utility of broadly targeting WM microstructure in the treatment for early-life anxiety. In this vein, recent work demonstrates that WM is remarkably plastic – particularly in youth – and responsive to various types of cognitive and motor training [67–70]. These studies lay the foundation for future research in clinical samples examining the extent to which existing cognitive and pharmacological therapies for anxiety may exert their effects in part by modulating WM microstructure. Studies in animal models suggest that stressors can impair myelination that is associated with reductions in social behavior [65, 71, 72]. Furthermore, pharmacological agents, including muscarinic antagonists such as clemastine and solifenacin, have been demonstrated to promote oligodendrocyte differentiation and enhance myelination [71, 73–75], and these effects are linked to the recovery of behavioral alterations [71, 73]. This pharmacological strategy could be used in conjunction with specific psychotherapeutic and/or training interventions that are targeted at maladaptive anxiety to potentially augment the reported effects of training and experience on WM integrity in pathways involved in mediating anxiety.

Although this study included a relatively large sample, most participants were White (Supplementary Table 4). Expanding the diversity of the participants in these studies to include more BIPOC individuals will be important to enhance generalizability of the findings. Our sample did not include boys, and our longitudinal analysis did not include control participants, limiting our conclusions regarding the association between WM microstructural integrity and anxiety symptoms to only girls with pathological anxiety. While there was some attrition in the follow-up years of the study, the statistical approaches used – linear mixed-effects models in particular – are designed to handle missing data in longitudinal datasets [60]. While we attribute the FA-anxiety relation to whole-brain reductions in FA, it is possible that there are associations between anxiety symptoms and FA in specific WM tracts that were not examined in this study.

In summary, we present one of the largest longitudinal neuroimaging studies of pediatric anxiety, demonstrating that, on an individual level in girls with pathological anxiety, worsening of anxiety symptoms is associated with a global decrease in WM microstructural integrity. Importantly, this relationship is independent of age and puberty. An extensive body of literature has shown that childhood and early adolescence are periods of significant WM growth across the brain [12, 76, 77]. Our present results demonstrate that within this overarching developmental pattern, individual variations in whole-brain WM are dynamically linked to childhood anxiety symptom severity. These findings support future studies investigating the possibility of targeting WM as a modality to aid in the prevention and treatment of childhood anxiety disorders.

## Supplementary information

Supplemental Materials

## Data Availability

Image processing and statistical code used for these studies, as well as imaging data, may be able to be shared with interested parties upon request by contacting the corresponding author.
