# Supplementary material for: A dynamic relation between whole-brain white matter microstructural integrity and anxiety symptoms in preadolescent females with pathological anxiety
Source: Transl Psychiatry. 2022 Feb 8;12:57. doi: 10.1038/s41398-022-01827-y (PMC8825837; doi:10.1038/s41398-022-01827-y)
Supplement: Supplementary file 1 — Supplemental Materials [file 41398_2022_1827_MOESM1_ESM.docx]

**Supplemental Materials for:**

**A Dynamic Relation Between Whole-Brain White Matter Microstructural Integrity and Anxiety Symptoms in Preadolescent Females with Pathological Anxiety**

Running Title:

**Longitudinal White Matter Alterations in Anxiety**

*Nakul Aggarwal, BS^1^; Lisa E. Williams, PhD^1^; Do P.M. Tromp, PhD^1^; Daniel S. Pine, MD^2^; Ned H. Kalin, MD^1^*

^1^Department of Psychiatry, University of Wisconsin-Madison, 6001 Research Park Boulevard, Madison, WI 53719, United States

^2^Section on Developmental and Affective Neuroscience, National Institute of Mental Health, Bethesda, MD 20814, United States

**Corresponding Author:**

Nakul Aggarwal

University of Wisconsin-Madison

Department of Psychiatry

6001 Research Park Boulevard

Madison, WI 53719

Email: naggarwal5@wisc.edu

Tel: 218-213-6910

**Supplementary Methods**

**DTI acquisition:** Images were collected on a 3.0 Tesla GE MR750 scanner (GE Healthcare; Waukesha, WI) using a 32-channel head coil. Diffusion-weighted MRI scans were obtained using a two-dimensional echo planar imaging diffusion-weighted spin-echo sequence (TR=6500ms, TE=59.6ms, flip angle=90 degrees, matrix=128x128 interpolated to 256x256, FOV=256mm, 2.9mm contiguous slices, echo-planar echo spacing=568μs, b-value of 1000s/mm^2^, 48 optimal non-collinear directions and 8 non-diffusion-weighted images). Structural and functional MRI scans were collected during the same scan sessions but are not reported here. Before each scan, children completed mock MRI sessions, which have been shown to reduce movement in pediatric neuroimaging studies [1].

**DTI processing and analysis:** Methods were comparable to those previously described in Tromp et al., 2019 [2]. Diffusion-weighted volumes from each individual at each scanning timepoint were transformed into a 3-dimensional diffusion tensor for each voxel in the brain using the following procedures. FSL [3] tools for rigid registration were used to correct distortions resulting from head motion and eddy currents [4]. The corresponding gradient direction matrix was corrected for the applied rotations after rigid registration. The brain was skull stripped using the FSL’s brain extraction tool [3]. Robust estimation of tensors by outlier rejection (RESTORE, as implemented in Camino software) [5] was used to minimize influence of noise on tensor calculation, a step particularly important in image samples of young/clinical populations that are more sensitive to reduced image quality. RESTORE uses an average noise estimation to determine which diffusion measurements are outliers and excludes those from tensor computation; it has been shown to increase the reliability of tensor estimation in clinical populations [6]. Resulting DTI scans contained 3 major vectors for each voxel in the brain that together model water diffusion as shaped by local tissue microstructure.

In order to compare diffusion measures across subjects and timepoints, scans were first normalized within-participant, followed by between-participant normalization of within-participant averages to create a study-specific template that was then, finally, warped to MNI-152 standard space. Individual tensor maps at each timepoint were generated in MNI space. These steps were performed using a high-dimensional registration method that incorporates tensor orientation (DTI-TK) [7], a technique that outperforms intensity-based normalization of diffusion images and results in improved white matter shape and architecture representation [8,9]. The final longitudinal population template was constructed via multiple registration iterations and then aligned to the 1mm isotropic MNI-152 template; this warp and the individual-to-participant average warp were then applied to all images. In MNI152 space, scalar maps for the primary DTI metric of interest, fractional anisotropy (FA), as well as mean diffusivity (MD) and radial diffusivity (RD), were calculated for each image.

The population template in standard MNI space, created from all participants’ data from all timepoints, was used for deterministic tractography to delineate tracts of interest. Whole-brain fiber tracking was performed using Camino software, which implements a fourth-order Runge-Kutta method combined with a tensor deflection (TEND) algorithm for optimal estimation of the fiber tracking directions [10,11]. Fiber tracking was terminated in voxels where FA was below 0.1 or where the angle between consecutive streamline steps was greater than 90 degrees. In addition to whole-brain WM, seven fiber pathways were iteratively delineated in template space using anatomically defined waypoints [12–15] in TrackVis, a 3D tract visualization program [16]. The 7 WM tracts of interest were selected based on substantial literature implicating alterations in these tracts in anxiety disorders and other internalizing disorders, in both adult and pediatric samples. In addition to the literature highlighted in relation to the UF, there is work suggesting other cortico-limbic association pathways may be affected in those with pathological anxiety and/or emotional dysregulation, including the cingulum bundle (CING) [17,18], the superior longitudinal fasciculus (SLF) [19,20], the fornix (FX) [21], and the inferior fronto-occipital fasciculus (IFO) [20,22]. Additionally, several publications have reported alterations in the projection fibers of the internal capsule (IC) [20,23] and commissural fibers of the corpus callosum (CC) [17,24] in patients with anxious and internalizing pathology. In turn, these 6 tracts, in addition to the UF, were included in our analysis.

In order to quantify the microstructure of entire white matter structures, weighted means were calculated per tract, per subject, per timepoint. The weighted mean of a tract was calculated by first creating a scalar image of the number of fibers in the tract passing through each voxel as a proportion of the total number of fibers in that tract. This weighting factor was then multiplied by the value of the diffusion measure in that voxel and averaged across the whole tract to produce the mean weighted scalar value for each tract [25]. This approach enables differential weighting of voxels that have higher fiber counts, observed frequently in areas more central to the white matter tract of interest. Importantly, tract-based analyses allow for detection of pervasive but subtle differences that are distributed across the length of a tract which may be missed using conventional voxel-based methods. In turn, this method is well-suited to identify tract-based associations in which alterations at any point in a tract might alter the efficiency of communication across a WM pathway.

**Bayesian Analyses:** Results from Bayesian analyses are reported as Bayes factors (BFs), which are symmetrical ratios that quantify the strength of the evidence the data provide for the alternative hypothesis (H_1_: anxiety and FA are related) over the null hypothesis (H_0_: anxiety and FA are unrelated), or vice versa [26]. For example, if an assessment of H_1_ compared to H_0_ yields a BF_H1_ of 5, that indicates the alternative hypothesis, H_1_, is 5 times more likely than the null hypothesis, H_0_, given the data. Conversely, in this same example, a BF_H1_ of 0.2 would indicate that the alternative hypothesis is 0.2 times more likely than the null hypothesis, or, in other words, that the null hypothesis is 5 times more likely than the alternative hypothesis (i.e., BF_H0_=5) – providing evidence for an absence of an effect. In our Bayesian ANCOVA and linear regression analyses, the null model predicted FA from age alone, while the alternative models predicted FA from both age and either group or child SCARED scores, respectively. BF_H1_ values between 1 and 3 are considered inconclusive (i.e., “absence of evidence”); between 3 and 10, moderate evidence of an effect; greater than 10, strong evidence of an effect. As BFs are symmetrical, conversely, BF_H1_ values between 0.33 and 1 are considered inconclusive; between 0.1 and 0.33, moderate evidence of an absence of an effect; less than 0.1, strong evidence of an absence of an effect [26]. Bayesian modeling was performed using JASP software (ver. 0.14.1).

**Linear Mixed-Effects (LME) Modeling:** LME models allow for precise and unbiased effect estimates by accounting for repeated within-participant measures [27]. Our primary LME models predicted FA (or MD or RD) from child SCARED scores, while covarying for age. All independent variables, including covariates, were mean-centered within-participant to enable estimation of within-participant effects [27]. In these models, in addition to the overall model intercept (β0), we estimate the fixed effect of child SCARED scores on FA (or MD or RD) within a participant (β1), while controlling for age. To account for the repeated longitudinal within-participant measurements, we also estimate three random effects: 1) the by-subject random intercept; 2) the by-subject random effect (slope) of child SCARED scores; and 3) the by-subject random effect (slope) of age. Lastly, these LME models also estimate the variance of the model residuals. All LME modeling was performed using the *lme4* package in RStudio (ver. 1.4.1106) [28].

**Supplementary Results**

**Child- and Parent-Rated SCARED Correlations:** In the cross-sectional analysis conducted in the full sample at study intake, child and parent SCARED scores were significantly correlated (between-participant correlation): *r*(174)=0.65, *P*<0.001. In the longitudinal analysis conducted in girls with pathological anxiety, child and parent SCARED scores were also significantly correlated (within-participant correlations): *r*(55.04)=0.18, *P*<0.001. Child SCARED scores were used as the primary metric of anxiety severity.

**Age, Tanner, and PDS Score Intercorrelations:** In the cross-sectional analysis conducted in the full sample at study intake, age, Tanner Staging Scores, and PDS scores were significantly inter-correlated (between-participant correlations): age-PDS: *r*(179)=0.53, *P*<0.001; age-Tanner: *r*(177)=0.57, *P*<0.001; PDS-Tanner: *r*(177)=0.80, *P*<0.001. In the longitudinal analysis conducted in girls with pathological anxiety, age, Tanner, and PDS were similarly inter-correlated (within-participant correlations): age-PDS: *r*(70.38)=0.52, *P*<0.001; age-Tanner: *r*(73.58)=0.52, *P*<0.001; PDS-Tanner: *r*(53.28)=0.55, *P*<0.001. In all analyses, age was used as the developmental covariate for all ANCOVA and regression models.

**Bayesian Analysis of Female Preadolescents in Tromp et al., 2019 (AJP) – UF FA: Between-Group ANCOVA (Controls vs. AD) and Cross-Sectional Linear Regression (with Child SCARED Scores):** To assess evidence of absence (or lack thereof) of an UF-anxiety relationship within the females included in our previously published sample [2], we performed a Bayesian analysis of the Tromp et al. 2019 sample of preadolescent females with and without ADs. Sample consists of 8–12-year-olds; 27 girls with ADs and 23 healthy controls girls. In contrast to the Bayesian UF results in the current sample, a between-group Bayesian ANCOVA in the Tromp et al. 2019 sample revealed absence of evidence (i.e., inconclusive evidence) for a group difference in UF FA (*F*(1,46)=0.30, *P*=0.587; **BF_H1_=0.335**). Similarly, a Bayesian linear regression of child SCARED scores against UF FA in the Tromp et al. 2019 sample also revealed absence of evidence for a relationship between anxiety and UF FA (*F*(1,44)=1.58, *P*=0.207; **BF_H1_=0.604**). Analyses controlled for age at scan and study site.

**Controlling for Depression (CDI) and Stressful Life Events (SLES) in the Primary SCARED-FA LME Model:** Depression and stressful life events were assessed by the Children’s Depression Inventory (CDI) and Stressful Life Events Schedule (SLES), respectively, in participants at every timepoint of participation. Because girls with severe depressive and trauma-related symptoms were excluded from study participation, depression and trauma load were relatively low (Table 1 in the manuscript). Depression was modeled as the CDI-Total T score. SLES was modeled as the number of child-reported stressful events (i.e., count). In addition to assessing the full SLES, to best capture trauma-specific events, we applied CDC definitions for adverse childhood events (ACEs; https://www.cdc.gov/violenceprevention/aces/about.html) – which revolve primarily around abuse, household challenges, and neglect – to select items from the full parent and child SLES surveys that best reflected trauma-related events, resulting in a subset of ~20 items for analysis. When including as within-participant covariates (log-transformed and mean-centered) in the primary model predicting whole-brain FA from SCARED scores and age, our original findings were unaffected, and a negative within-participant relation between SCARED scores and whole-brain FA remained, independent of changes in depression (CDI) or SLES-ACE counts. (with CDI: Std. β (95% CI)=-0.08 (-0.10 to -0.06), *F*(1,40.62)=10.29, *P*=0.003; with SLES-ACE: Std. β (95% CI)=-0.06 (-0.08 to -0.04), *F*(1,43.91)=8.79, *P*=0.005).


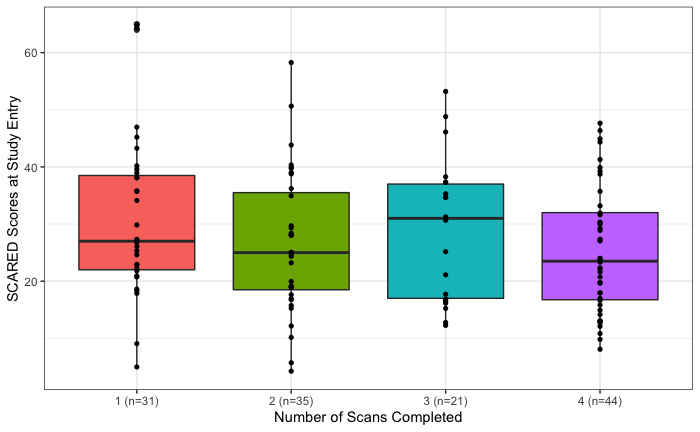


To assess whether anxiety severity at study entry was related to sample attrition in girls with pathological anxiety that were followed longitudinally, we performed a one-way ANCOVA comparing child SCARED scores at study entry across four groups: 1) participants with 1 total scan, 2) 2 total scans, 3) 3 total scans, or 4) 4 total scans (i.e., completed the study). This analysis controlled for age at study entry. ANCOVA results (visualized in the boxplot graph) indicated no significant differences between any two groups (*F*(3,126)=1.08, *P*=0.359).

**Supplementary Figure 1. Comparison of Child SCARED Scores at Study Entry Among Participants with 1, 2, 3, or 4 Total Scans**


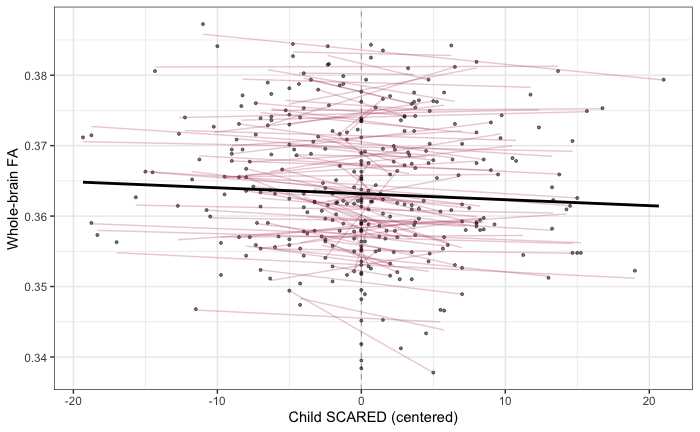


Within-participant relationship between whole-brain FA and child SCARED scores, while controlling for pubertal status (PDS scores) instead of age (Std. β (95% CI)=-0.06 (-0.09 to -0.03), *F*(1,43.78)=11.45, *P*=0.002). Each maroon line represents a participant-specific regression line predicting whole-brain FA from within-participant centered child SCARED scores, while controlling for PDS scores. Each point represents an individual scan. The bolded black line in each graph depicts the average within-participant association of whole-brain FA with child SCARED scores.

**Supplementary Figure 2. Longitudinal Within-Participant Association of Child SCARED Scores with Whole-Brain FA while Controlling for PDS Scores (in lieu of Age)**

**Supplementary Figure 3. Cross-Sectional Between-Participant Association of Age with Whole-Brain FA at Study Entry**


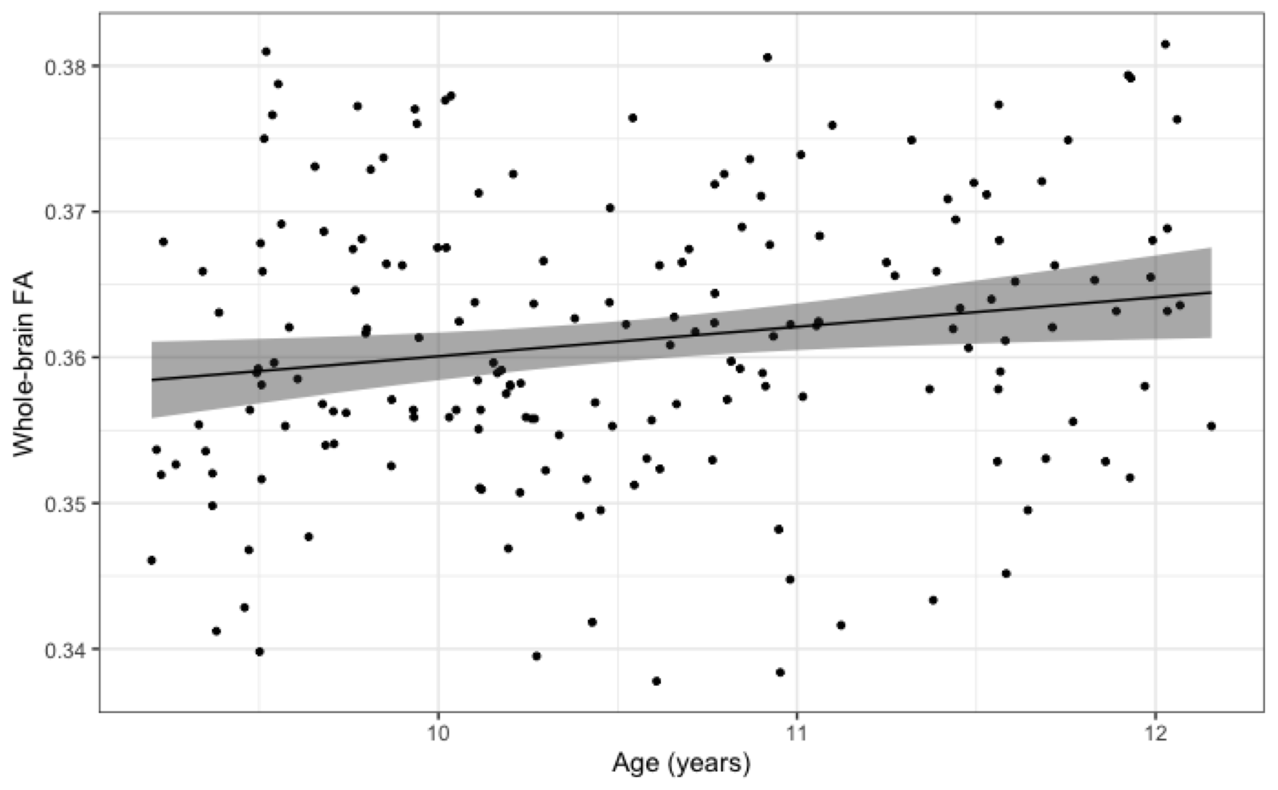


At study entry (n=182), there is a significant positive cross-sectional relationship between age and whole-brain FA (Std. β (95% CI)=0.08 (0.05 to 0.12), *F*(1,64.90)=24.75, *P*<0.001).

**Supplementary Figure 4. Longitudinal Within-Participant Association of Child SCARED Scores with Whole-Brain MD and RD**


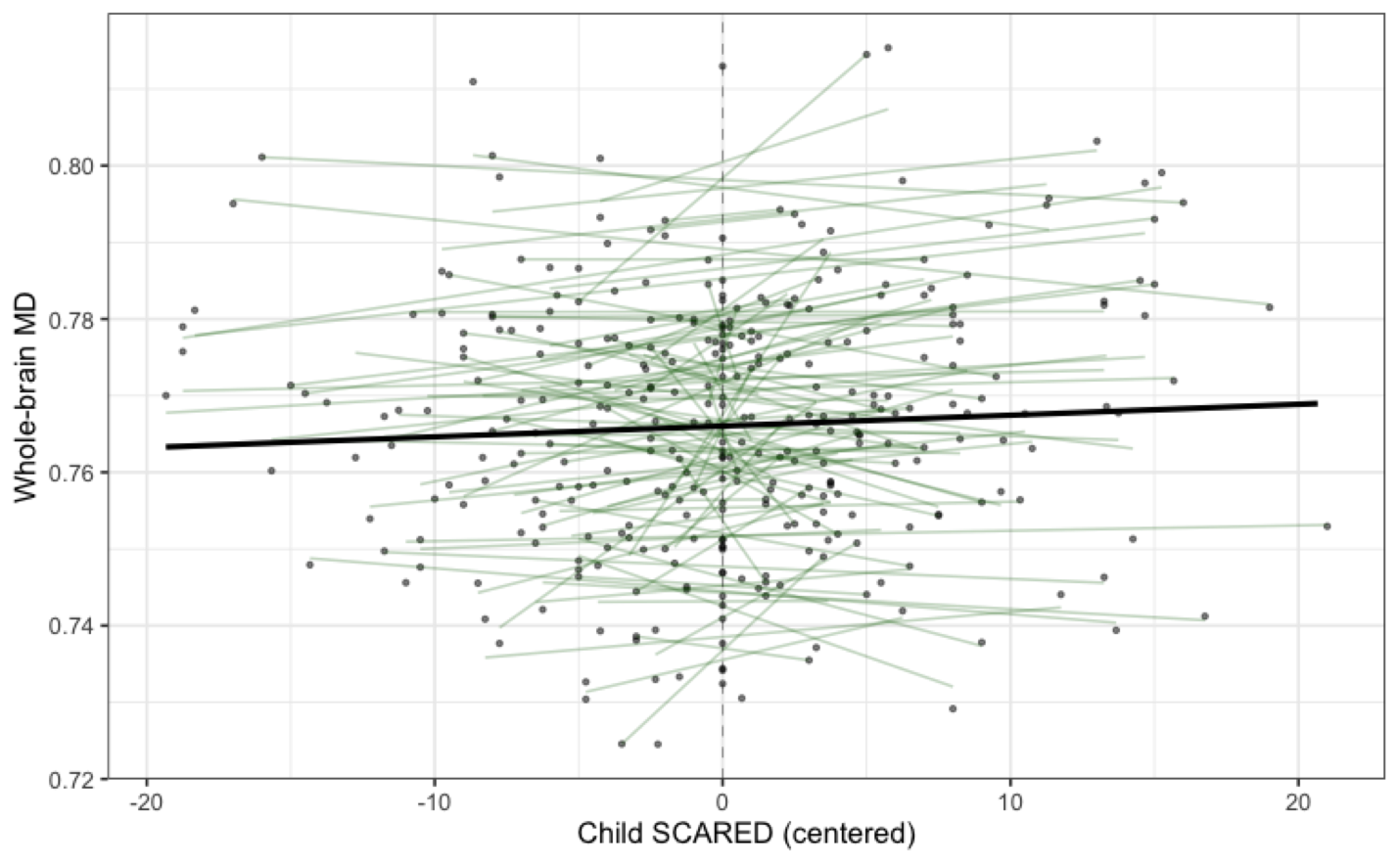


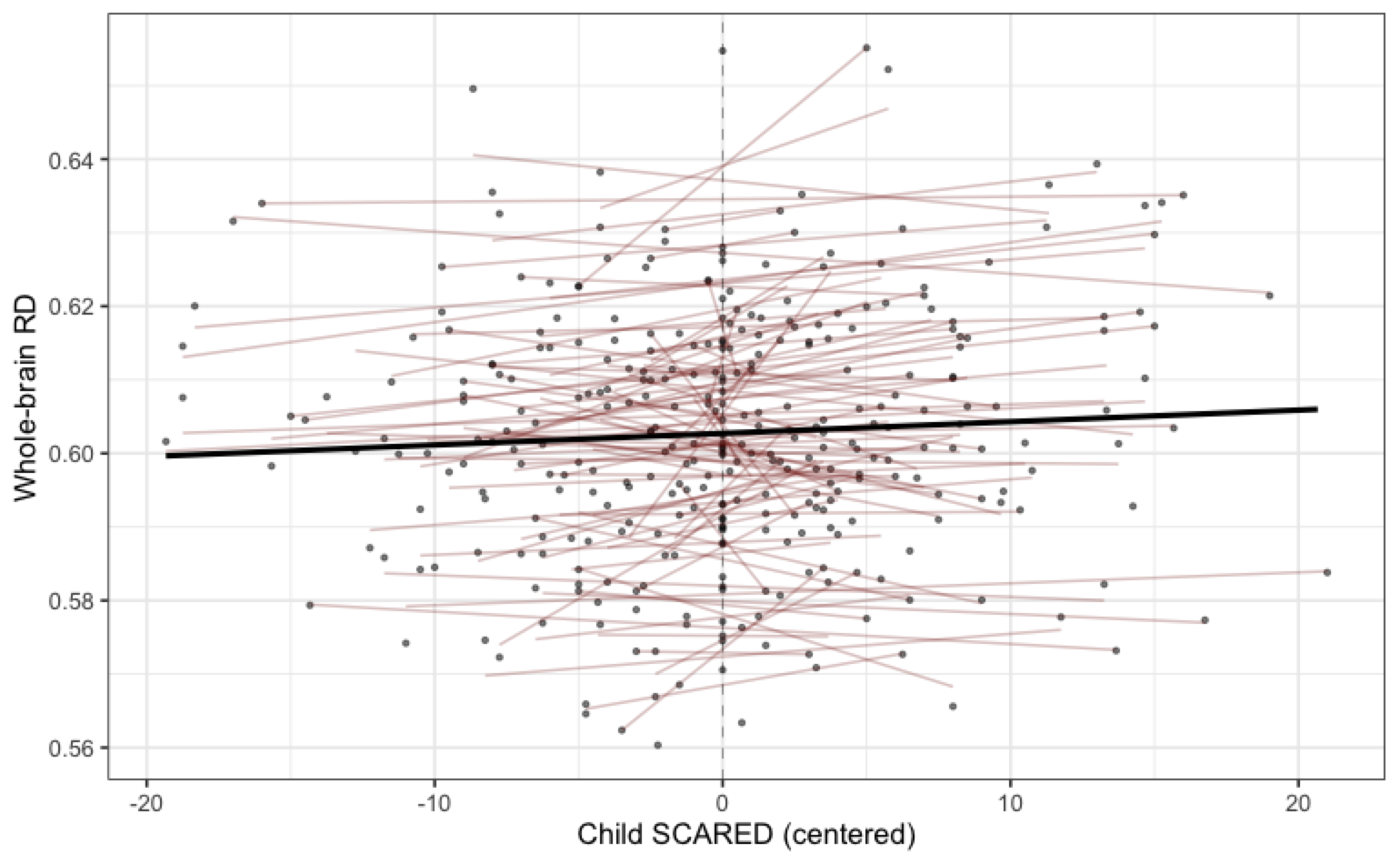


Within-participant relationship between whole-brain MD (top) and RD (bottom) and child SCARED scores. MD and RD are thought to be more specific to myelination and axonal density and are generally inversely related to FA (i.e., higher MD and RD values indicate reduced WM integrity). Each green or red line represents a participant-specific regression line predicting whole-brain MD (Std. β (95% CI)=0.06 (0.02 to 0.11), *F*(1,44.76)=4.12, *P*=0.048) or RD (Std. β (95% CI)=0.06 (0.01 to 0.11), *F*(1,44.99)=6.09, *P*=0.017), respectively, from within-participant centered child SCARED scores, while controlling for age. Each point represents an individual scan. The bolded black line in each graph depicts the average within-participant association of whole-brain MD or RD with child SCARED scores. As expected, MD and RD exhibit positive associations with child SCARED scores, but these do not survive multiple comparison correction.

**Supplementary Figure 5. Longitudinal Within-Participant Association of Parent SCARED Scores with Whole-Brain FA**


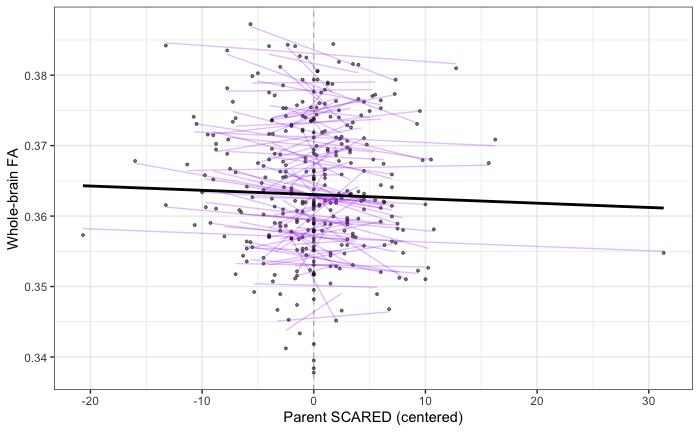

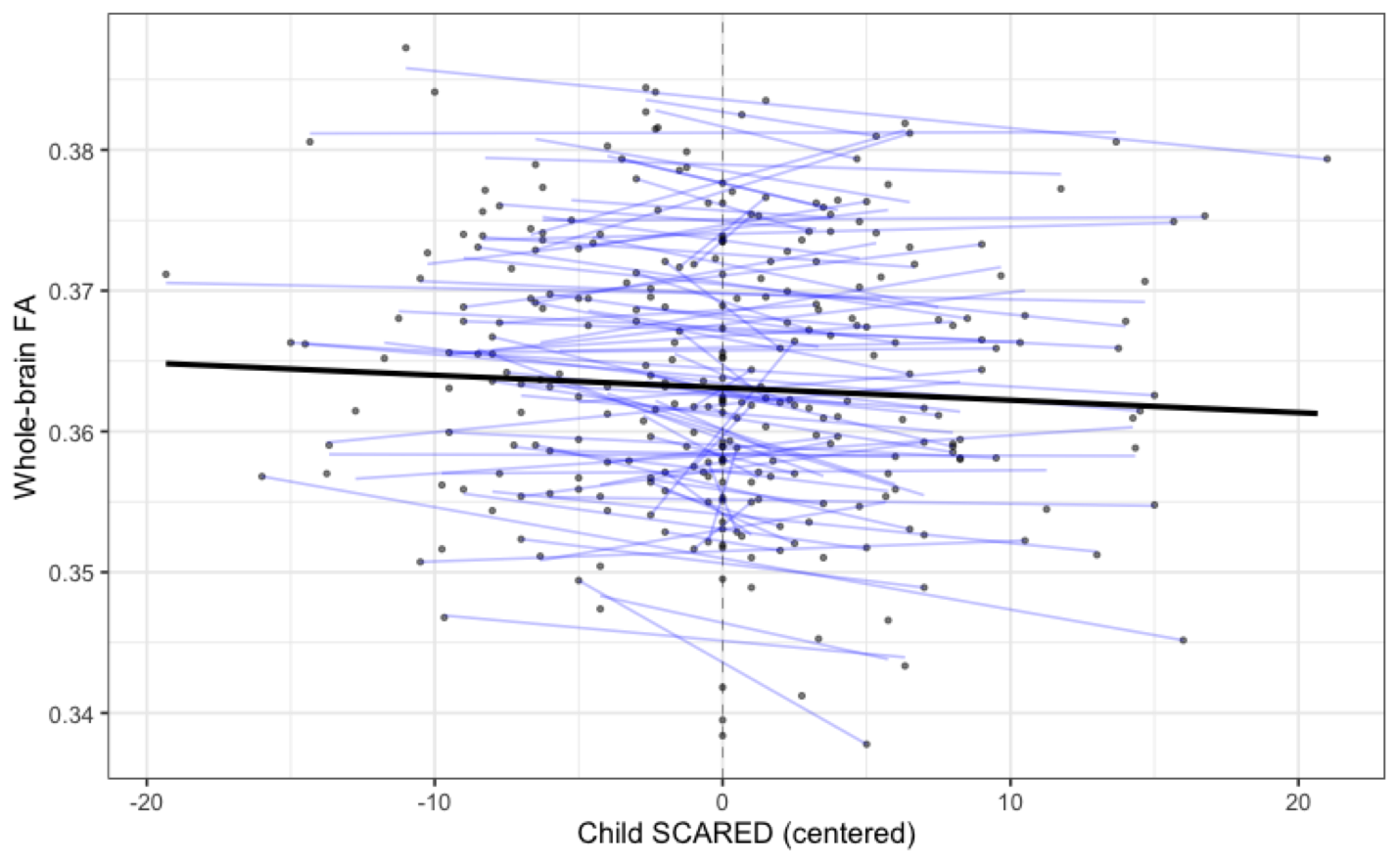


To assess how parent SCARED scores related to whole-brain FA on an individual level, in a supplemental analysis, child SCARED scores were replaced with parent SCARED in our main linear mixed-effects model (while still controlling for age). This analysis was performed on the sample of observations with complete DTI-parent SCARED paired data (n=132 participants; n=325 observations).

Similar to the finding with child SCARED scores (Figure 2 in the main text), a negative within-participant relationship between whole-brain FA and parent SCARED scores was also observed (Std. β (95% CI)=-0.04 (-0.07 to 0.00), *F*(1,17.56)=1.98, *P*=0.177); however it was not statistically significant. Each purple line represents a participant-specific regression line predicting whole-brain FA from within-participant centered parent SCARED scores, while controlling for age. Each point represents an individual scan. The bolded black line in each graph depicts the average within-participant association of whole-brain FA with parent SCARED scores.

**Supplementary Figure 6. Longitudinal Within-Participant Association of Child SCARED Scores with Whole-Brain FA in Treatment-Free Sample Subset**

To examine whether treatment during study participation impacted the results reported here, in a supplemental analysis, scans collected after treatment initiation were excluded. Consistent with the findings from the full sample, this analysis revealed a significant relationship between whole-brain FA and anxiety after multiple comparison correction (*F*(1,43.13)=9.86, *P*=0.003).

Within-participant relationship between whole-brain FA and child SCARED scores in treatment-free subset of data. Of 133 subjects, 27 received behavioral or pharmacological therapy for anxiety at some point in the study. Data collected from participants after the start of treatment were excluded in this analysis, resulting in the exclusion of 41 data points (out of 343). Each blue line represents a participant-specific regression line predicting whole-brain FA from within-participant centered child SCARED scores, while controlling for age. Each point represents an individual scan. The bolded black line depicts the average within-participant association of whole-brain FA with child SCARED scores.

**Supplementary Figure 7. Longitudinal Within-Participant Association of Age with Whole-Brain FA and Child SCARED Scores**


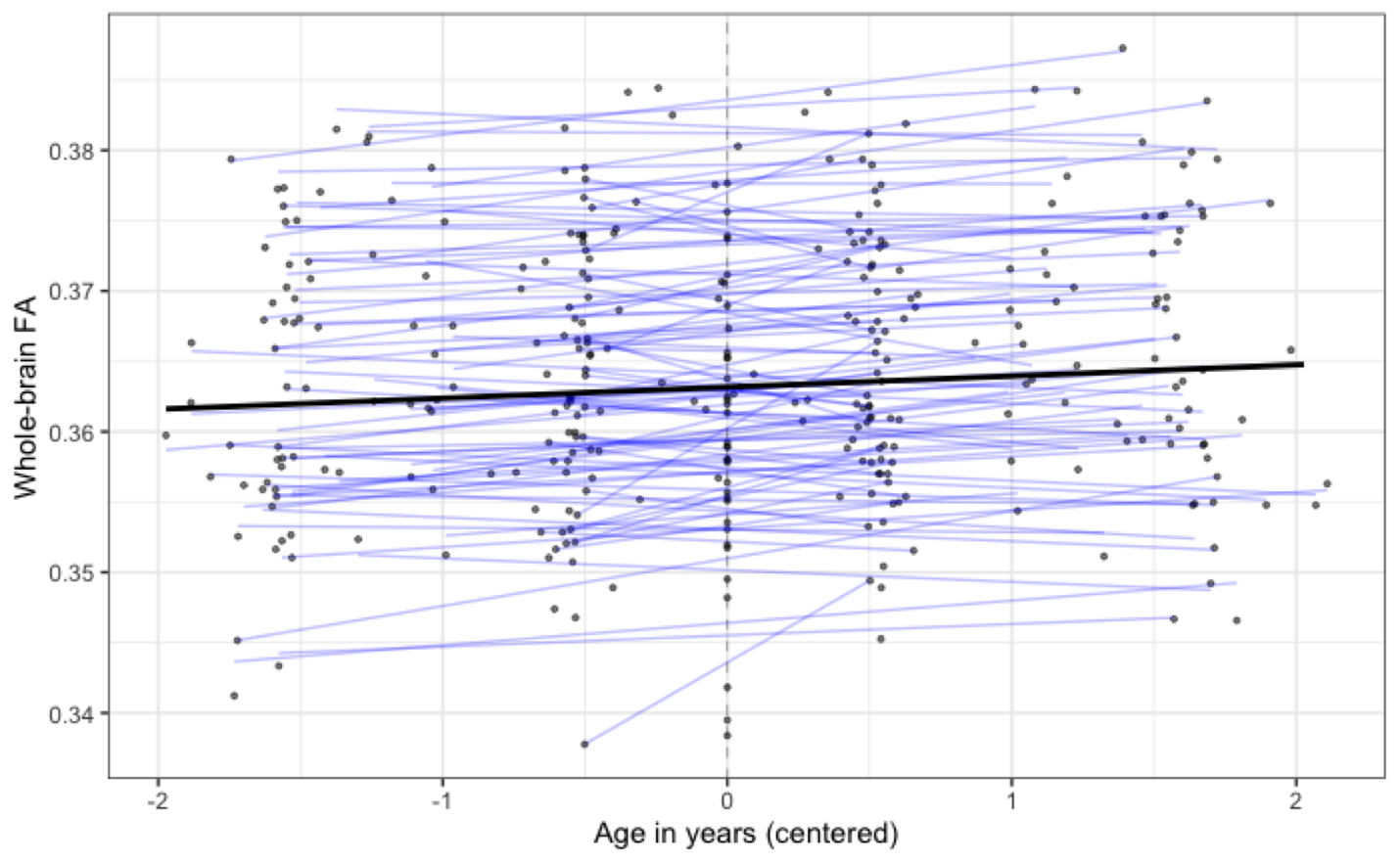


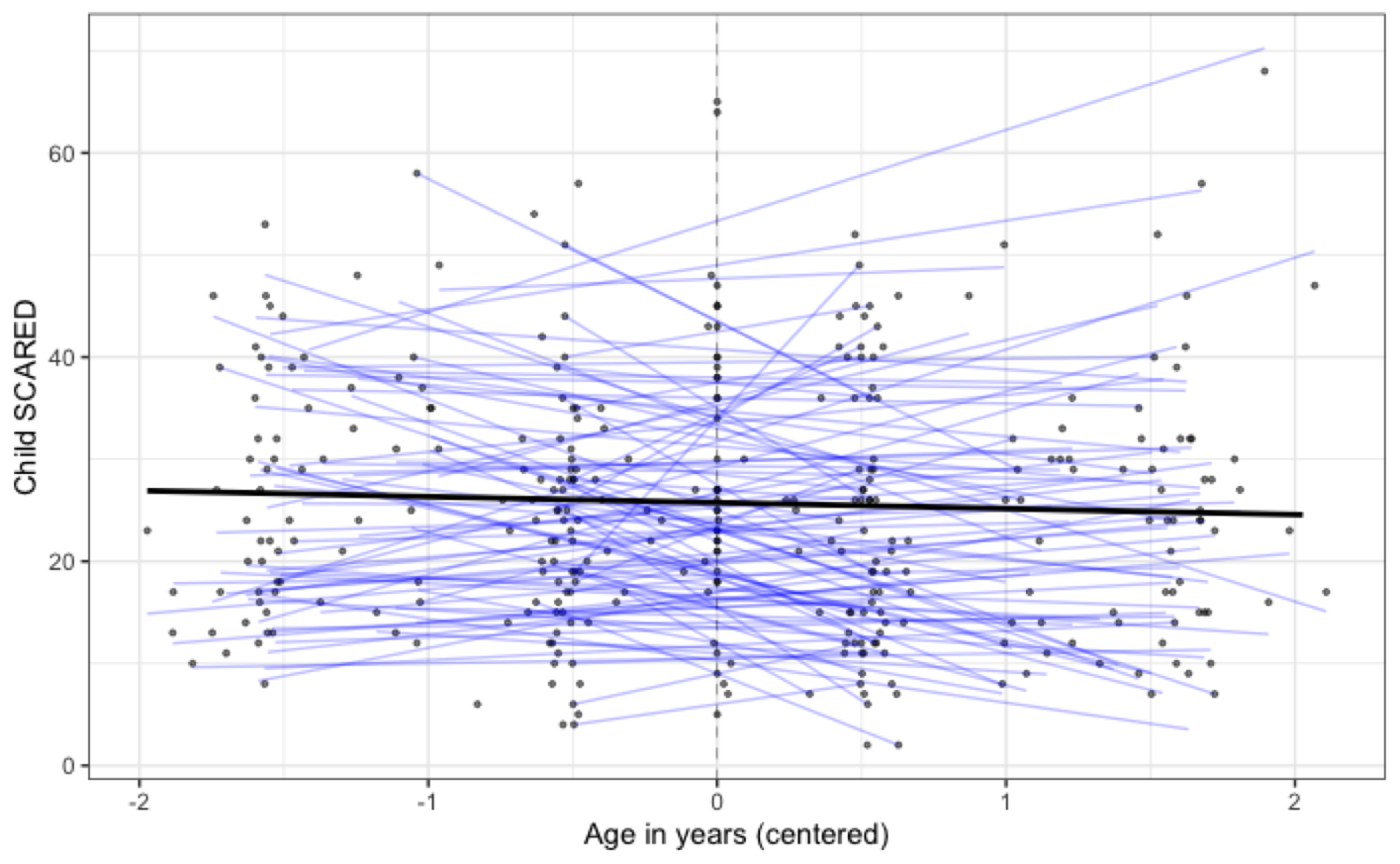


To 1) confirm developmental increases in WM microstructural integrity and 2) assess whether anxiety was related to age in our sample, we performed two linear mixed-effects analyses to obtain the within-participant associations between 1) age and whole-brain, and 2) age and child SCARED scores.

Within-participant relationship between age and whole-brain FA (top) (Std. β (95% CI)=0.08 (0.05 to 0.12), *F*(1,64.90)=24.75, *P*<0.001) and child SCARED scores (bottom) (Std. β (95% CI)=-0.05 (-0.14 to 0.04), *F*(1,78.09)=1.04, *P*=0.311). Each blue line represents a participant-specific regression line predicting whole-brain FA or child SCARED scores from within-participant centered age (in years). Each point represents an individual scan or questionnaire timepoint. The bolded black line in each graph depicts the average within-participant association of age with whole-brain FA or child SCARED scores.

Between-participant relationships between age and pubertal status (PDS) with whole-brain and tract-specific FA at study entry. Raw and standardized beta estimates (effect sizes) and P-values are listed for each association. On a between-participant level, as expected, FA increases with age and pubertal status across the brain.

| **Supplementary Table 1. Linear Regression Statistics for Cross-Sectional Between-Participant Associations of Age and Pubertal Status with Whole-Brain and Tract-Specific FA at Study Entry** | | | | | | |
| --- | --- | --- | --- | --- | --- | --- |
|  | **Age** | | | **PDS** | | |
| **Bilateral WM Tract** | **β1** | **Std. β1 (95% CI)** | ***P* value** | **β1** | **Std. β1 (95% CI)** | ***P* value** |
| WB | 1.749e-03 | 0.15 (0.00 to 0.30) | 0.046 | 2.710e-03 | 0.16 (0.01 to 0.31) | 0.040 |
| CC | 1.459e-03 | 0.09 (-0.06 to 0.24) | 0.260 | 2.426e-03 | 0.10 (-0.06 to 0.25) | 0.215 |
| CING | 0.0060335 | 0.24 (0.10 to 0.39) | 0.001 | 0.0063481 | 0.17 (0.02 to 0.32) | 0.024 |
| IC | 3.040e-03 | 0.20 (0.06 to 0.35) | 0.007 | 4.354e-03 | 0.19 (0.05 to 0.34) | 0.011 |
| IFO | 3.195e-03 | 0.19 (0.04 to 0.34) | 0.013 | 4.301e-03 | 0.17 (0.02 to 0.32) | 0.028 |
| SLF | 9.160e-04 | 0.05 (-0.10 to 0.19) | 0.544 | 3.007e-03 | 0.10 (-0.05 to 0.25) | 0.186 |
| STRIA/FX | -2.377e-04 | -0.01 (-0.16 to 0.14) | 0.868 | 5.764e-04 | 0.02 (-0.13 to 0.17) | 0.790 |
| UF | 2.615e-03 | 0.16 (0.01 to 0.31) | 0.032 | 4.879e-03 | 0.20 (0.05 to 0.35) | 0.008 |

| **Supplementary Table 2. Average Within-Participant Associations Between SCARED Scores and Tract MD and RD** | | | | | | | | |
| --- | --- | --- | --- | --- | --- | --- | --- | --- |
|  | **MD** | | | | **RD** | | | |
| **Bilateral WM Tract** | **β1** | **Std. β1 (95% CI)** | **P value** | **β1** | | **Std. β1 (95% CI)** | **P value** |  |
| WB | 1.415e-04 | 0.06 (0.01 to 0.11) | 0.048 | 0.0001581 | | 0.06 (0.02 to 0.11) | 0.017 |  |
| CC | 1.222e-04 | 0.04 (0.00 to 0.08) | 0.106 | 1.572e-04 | | 0.05 (0.01 to 0.08) | 0.031 |  |
| CING | 1.786e-04 | 0.06 (0.02 to 0.11) | 0.018 | 2.082e-04 | | 0.06 (0.02 to 0.09) | 0.004 |  |
| IC | 1.235e-04 | 0.05 (-0.03 to 0.12) | 0.243 | 1.147e-04 | | 0.04 (-0.02 to 0.10) | 0.206 |  |
| IFO | 7.936e-05 | 0.03 (-0.01 to 0.07) | 0.192 | 1.279e-04 | | 0.04 (0.01 to 0.08) | 0.029 |  |
| SLF | 1.066e-04 | 0.04 (0.00 to 0.09) | 0.074 | 1.264e-04 | | 0.04 (0.01 to 0.08) | 0.024 |  |
| STRIA/FX | 0.0005669 | 0.07 (0.02 to 0.12) | 0.009 | 0.0005664 | | 0.07 (0.02 to 0.12) | 0.00629 |  |
| UF | 5.503e-05 | 0.02 (-0.03 to 0.07) | 0.481 | 7.387e-05 | | 0.03 (-0.02 to 0.07) | 0.343 |  |

While whole-brain MD and RD were not significantly associated with anxiety severity after multiple comparison correction, positive within-participant correlations were observed between child SCARED scores and both MD (*F*(1,44.76)=4.12, *P*=0.048) and RD (*F*(1,44.99)=6.09, *P*=0.017), DTI metrics thought to be more specific to myelination and axonal density.

| **Supplementary Table 3. Linear Mixed-Effects Model Statistics for Longitudinal Within-Participant Associations of Age and Pubertal Status with Whole-Brain and Tract-Specific FA** | | | | | | |
| --- | --- | --- | --- | --- | --- | --- |
|  | **Age** | | | **PDS** | | |
| **Bilateral WM Tract** | **β1** | **Std. β1 (95% CI)** | ***P* value** | **β1** | **Std. β1 (95% CI)** | ***P* value** |
| WB | 7.893e-04 | 0.08 (0.05 to 0.12) | <0.001 | 1.325e-03 | 0.07 (0.03 to 0.10) | <0.001 |
| CC | 8.716e-04 | 0.06 (0.03 to 0.10) | <0.001 | 1.360e-03 | 0.05 (0.01 to 0.08) | 0.013 |
| CING | 3.352e-03 | 0.16 (0.13 to 0.18) | <0.001 | 5.648e-03 | 0.12 (0.10 to 0.15) | <0.001 |
| IC | 1.502e-03 | 0.12 (0.08 to 0.16) | <0.001 | 2.776e-03 | 0.10 (0.06 to 0.14) | <0.001 |
| IFO | 1.905e-03 | 0.13 (0.09 to 0.16) | <0.001 | 0.0027389 | 0.09 (0.05 to 0.12) | <0.001 |
| SLF | 4.479e-04 | 0.03 (0.00 to 0.06) | 0.079 | 1.766e-04 | 0.01 (-0.03 to 0.04) | 0.756 |
| STRIA/FX | -2.368e-03 | -0.15 (-0.19 to -0.11) | <0.001 | -3.974e-03 | -0.12 (-0.16 to -0.08) | <0.001 |
| UF | -4.675e-05 | -0.00 (-0.04 to 0.04) | 0.885 | -5.450e-04 | -0.02 (-0.06 to 0.02) | 0.421 |

Within-participant relationships between age and pubertal status (PDS) with whole-brain and tract-specific FA. Raw and standardized beta estimates (effect sizes) and P-values are listed for each association. On a within-participant level, similar to between-participant shown above, FA increases with both age and pubertal status.

| **Supplementary Table 4. Racial Breakdown by Cohort** | | | | |
| --- | --- | --- | --- | --- |
|  | **Group** | | |  |
| **Race, count (% of total sample)** | **Healthy Control** | **Subthreshold-AD** | **AD** | **Total** |
| Asian | 1 (0.55) | 0 (0) | 0 (0) | 1 (0.55) |
| Black | 2 (1.01) | 3 (1.65) | 1 (0.55) | 6 (3.30) |
| Native American | 0 (0) | 2 (1.01) | 0 (0) | 2 (1.01) |
| White | 40 (21.98) | 69 (37.91) | 41 (22.53) | 150 (82.42) |
| Multi-Racial | 5 (2.75) | 7 (3.85) | 9 (4.95) | 21 (11.54) |
| Not Provided | 1 (0.55) | 1 (0.55) | 0 (0) | 2 (1.01) |
| **Total** | 49 (26.92) | 82 (45.06) | 51 (28.02) | 182 (100) |

Racial breakdown of the sample by cohort at study entry. A chi-square test revealed no differences in racial distributions among the three groups (χ^2^ (10, n=182)=8.95, *P*=0.537).

**REFERENCES**

1. Bie HMA de, Boersma M, Wattjes MP, Adriaanse S, Vermeulen RJ, Oostrom KJ, et al. Preparing children with a mock scanner training protocol results in high quality structural and functional MRI scans. Eur J Pediatr. 2010;169(9):1079–85.

2. Tromp DPM, Williams LE, Fox AS, Oler JA, Roseboom PH, Rogers GM, et al. Altered Uncinate Fasciculus Microstructure in Childhood Anxiety Disorders in Boys But Not Girls. American Journal of Psychiatry. 2019 Mar;176(3):208–16.

3. Jenkinson M, Beckmann CF, Behrens TEJ, Woolrich MW, Smith SM. FSL. Neuroimage. 2012;62(2):782–90.

4. Leemans A, Jones DK. The B-matrix must be rotated when correcting for subject motion in DTI data. Magnet Reson Med. 2009;61(6):1336–49.

5. Camino: Open-Source Diffusion-MRI Reconstruction and Processing - 02759.pdf [Internet]. [cited 2021 Jun 24]. Available from: https://afni.nimh.nih.gov/sscc/staff/rwcox/ISMRM_2006/ISMRM%202006%20-%203340/files/02759.pdf

6. Chang L-C, Jones DK, Pierpaoli C. RESTORE: Robust estimation of tensors by outlier rejection. Magnet Reson Med. 2005;53(5):1088–95.

7. Zhang H, Yushkevich P, Alexander D, Gee J. Deformable registration of diffusion tensor MR images with explicit orientation optimization. Med Image Anal. 2006;10(5):764–85.

8. Zhang H, Avants BB, Yushkevich PA, Woo JH, Wang S, McCluskey LF, et al. High-Dimensional Spatial Normalization of Diffusion Tensor Images Improves the Detection of White Matter Differences: An Example Study Using Amyotrophic Lateral Sclerosis. Ieee T Med Imaging. 2007;26(11):1585–97.

9. Adluru N, Zhang H, Fox AS, Shelton SE, Ennis CM, Bartosic AM, et al. A diffusion tensor brain template for rhesus macaques. Neuroimage. 2011;59(1):306–18.

10. Basser PJ, Pajevic S, Pierpaoli C, Duda J, Aldroubi A. In vivo fiber tractography using DT-MRI data. Magnet Reson Med. 2000;44(4):625–32.

11. Lazar M, Weinstein DM, Tsuruda JS, Hasan KM, Arfanakis K, Meyerand ME, et al. White matter tractography using diffusion tensor deflection. Hum Brain Mapp. 2003;18(4):306–21.

12. Catani M, Howard RJ, Pajevic S, Jones DK. Virtual in vivo interactive dissection of white matter fasciculi in the human brain. Neuroimage. 2002;17(1):77–94.

13. Catani M, Thiebautdeschotten M. A diffusion tensor imaging tractography atlas for virtual in vivo dissections. Cortex. 2008;44(8):1105–32.

14. Mori S, Kaufmann WE, Davatzikos C, Stieltjes B, Amodei L, Fredericksen K, et al. Imaging cortical association tracts in the human brain using diffusion-tensor-based axonal tracking. Magnet Reson Med. 2002;47(2):215–23.

15. Wakana S, Jiang H, Nagae-Poetscher LM, Zijl PCM van, Mori S. Fiber tract-based atlas of human white matter anatomy. Radiology. 2003;230(1):77–87.

16. TrackVis [Internet]. [cited 2021 Jul 15]. Available from: http://trackvis.org/

17. Hung Y, Uchida M, Gaillard SL, Woodworth H, Kelberman C, Capella J, et al. Cingulum-Callosal White-Matter Microstructure Associated with Emotional Dysregulation in Children: A Diffusion Tensor Imaging Study. Neuroimage Clin. 2020;27:102266.

18. Zhang Y, Li L, Yu R, Liu J, Tang J, Tan L, et al. White matter integrity alterations in first episode, treatment-naive generalized anxiety disorder. J Affect Disorders. 2013;148(2–3):196–201.

19. Baur V, Hänggi J, Rufer M, Delsignore A, Jäncke L, Herwig U, et al. White matter alterations in social anxiety disorder. J Psychiatr Res. 2011;45(10):1366–72.

20. Liao M, Yang F, Zhang Y, He Z, Su L, Li L. White matter abnormalities in adolescents with generalized anxiety disorder: a diffusion tensor imaging study. Bmc Psychiatry. 2014;14(1):41.

21. Modi S, Trivedi R, Singh K, Kumar P, Rathore RKS, Tripathi RP, et al. Individual differences in trait anxiety are associated with white matter tract integrity in fornix and uncinate fasciculus: Preliminary evidence from a DTI based tractography study. Behav Brain Res. 2013;238:188–92.

22. Lai C-H, Wu Y-T. Fronto-occipital fasciculus, corpus callosum and superior longitudinal fasciculus tract alterations of first-episode, medication-naïve and late-onset panic disorder patients. J Affect Disorders. 2013;146(3):378–82.

23. Hyett MP, Perry A, Breakspear M, Wen W, Parker GB. White matter alterations in the internal capsule and psychomotor impairment in melancholic depression. Plos One. 2018;13(4):e0195672.

24. Westlye LT, Bjørnebekk A, Grydeland H, Fjell AM, Walhovd KB. Linking an Anxiety-Related Personality Trait to Brain White Matter Microstructure: Diffusion Tensor Imaging and Harm Avoidance. Arch Gen Psychiat. 2011;68(4):369–77.

25. Tromp D. Calculate tract based weighted means [Internet]. [cited 2021 Jul 15]. Available from: https://www.authorea.com/users/226778/articles/284738-calculate-tract-based-weighted-means?commit=466a6f14b3577bd8592ab0b000b8073fd71a88e9

26. Keysers C, Gazzola V, Wagenmakers E-J. Using Bayes factor hypothesis testing in neuroscience to establish evidence of absence. Nat Neurosci. 2020;23(7):788–99.

27. Brauer M, Curtin JJ. Linear Mixed-Effects Models and the Analysis of Nonindependent Data: A Unified Framework to Analyze Categorical and Continuous Independent Variables that Vary Within-Subjects and/or Within-Items. Psychol Methods. 2018;23(3):389–411.

28. Bates D, Mächler M, Bolker B, Walker S. Fitting Linear Mixed-Effects Models Using lme4. J Stat Softw. 2015;67(1).
